# On Plasters Containing Caoutchouc

**Published:** 1890-08

**Authors:** Grover William Wende

**Affiliations:** Acting Assistant Surgeon, Soldiers’ Home, Bath, N. Y.


### ON PLASTERS CONTAINING CAOUTCHOUC.

By Drs. SCHNEEGANS AND CORNEILLE.

Translated from the *Pharmaceutische Zeitung* for the Buffalo Medical and Surgical Journal.

By GROVER WILLIAM WENDE, M. D.,

Acting Assistant Surgeon, Soldiers' Home, Bath, N. Y.

The mass which we employ as a base for our plasters consists of lanoline, benzoinated tallow, caoutchouc, and dammar gum in varying proportions. Just enough of caoutchouc and gum are added to render them sufficiently adhesive, the greater part of the mass being composed of lanoline and fat, which impart to the skin a non-irritating effect. The addition of a small amount of glycerine will prevent drying and cracking on exposure to the air. The caoutchouc is added to the mass in a solution of benzine, 1-6. The solution is easily made by macerating rolled caoutchouc as ordinarily found in the market, in six times its weight of benzine, to be shaken frequently. At first the caoutchouc swells, but in the course of three or four days it will be completely dissolved.

OXIDE OF ZINC PLASTERS.—20 per cent.

| | |
|---|---|
| ℞—Resin Dammar | 15 parts. |
| Seb. Benzoinat | 25 " |
| Lanolini | 15 " |
| Caoutchouc | 5 " |
| Glycerine | 20 " |
| Zinci Oxidi | 20 " |
| | 100 parts. |

After the resin has been melted upon an open fire, the tallow is added. The whole is strained through muslin folded three or four times. On being continually stirred, to this is added the lanoline and the solution of caoutchouc. By this method such a uniform mixture is obtained that the necessary precautions must be observed in bringing it upon the water-bath, owing to the evaporation of the benzine.

In working with larger quantities, the benzine may be abstracted and used anew. When the benzine has been entirely evaporated, the oxide of zinc, well rubbed up in glycerine, is next put in. The whole is thoroughly worked until the mass becomes homogeneous. It is now allowed to stand in a warm place till the air-bubbles cease to escape from the compound, then it is spread over shirting by means of a plaster machine. It is essential not to use the mass too warm, as it is apt to soak through the cloth. It is better to wait until it arrives at a proper consistency and then warm the machine slightly. The thickness of the layer will vary from that of an ordinary sheet of writing paper to that of a common playing card. With a little practice an individual will be able in the course of an hour to an hour and a half to use conveniently about two pounds of the mass of the above prescription, giving him about thirty-six yards of plaster eight inches wide.

The plaster is allowed to dry for two or three days in the open air, after which it is covered with muslin. Paper will answer to preserve this preparation, as the glycerine prevents further drying.

IODOFORM PLASTER.—20 per cent.

| | |
|---|---|
| ℞—Resin Dammar | 15 parts. |
| Seb. Benzoin | 30 " |
| Lanolini | 20 " |
| Caoutchouc | 5 " |
| Glycerine | 10 " |
| Iodoformi | 20 " |
| | 100 parts. |

The process in preparing the iodoform plaster is similar to the one above described. It is necessary, however, to add the iodoform, well rubbed up in glycerine, to the mass after it has cooled off in order to prevent the evaporation of the iodoform. This plaster is best preserved in tin boxes.

MERCURIAL PLASTER.—20 per cent.

| | |
|---|---|
| ℞—Resin Dammar | 20 parts. |
| Seb. Benzoin | 34 " |
| Lanolini | 20 " |
| Caoutchouc | 6 " |
| Hydrarg. viv | 20 " |
| | 100 parts. |

The mercury and lanoline are well rubbed together until no particles of mercury are visible. The color is at first a light gray; during the process of mixing it becomes darker, and finally of a bluish gray. As heretofore, the resin and tallow are melted together, passed through muslin, the solution of caoutchouc added, and the benzine evaporated on the water-bath. To this, when still moderately warm, the mercury and lanoline are added and thoroughly worked. It is allowed to remain at a low heat till all air-bubbles have escaped. When in a semi-fluid condition, it is passed through the plaster machine. This plaster is best kept in tin boxes and in a cool place. It is of a rather soft consistence during the summer weather. The formula may be modified accordingly.

